# Cortical Modulations Increase in Early Sessions with Brain-Machine Interface

**DOI:** 10.1371/journal.pone.0000619

**Published:** 2007-07-18

**Authors:** Miriam Zacksenhouse, Mikhail A. Lebedev, Jose M. Carmena, Joseph E. O'Doherty, Craig Henriquez, Miguel A.L. Nicolelis

**Affiliations:** 1 Faculty of Mechanical Engineering, Technion, Haifa, Israel; 2 Department of Neurobiology, Duke University, Durham, North Carolina, United States of America; 3 Center for Neuro-engineering, Duke University, Durham, North Carolina, United States of America; 4 Department of Biomedical Engineering, Duke University, Durham, North Carolina, United States of America; 5 Department of Psychological and Brain Sciences, Duke University, Durham, North Carolina, United States of America; Indiana University, United States of America

## Abstract

**Background:**

During planning and execution of reaching movements, the activity of cortical motor neurons is modulated by a diversity of motor, sensory, and cognitive signals. Brain-machine interfaces (BMIs) extract part of these modulations to directly control artificial actuators. However, cortical modulations that emerge in the novel context of operating the BMI are poorly understood.

**Methodology/Principal Findings:**

Here we analyzed the changes in neuronal modulations that occurred in different cortical motor areas as monkeys learned to use a BMI to control reaching movements. Using spike-train analysis methods we demonstrate that the modulations of the firing-rates of cortical neurons increased abruptly after the monkeys started operating the BMI. Regression analysis revealed that these enhanced modulations were not correlated with the kinematics of the movement. The initial enhancement in firing rate modulations declined gradually with subsequent training in parallel with the improvement in behavioral performance.

**Conclusions/Significance:**

We conclude that the enhanced modulations are related to computational tasks that are significant especially in novel motor contexts. Although the function and neuronal mechanism of the enhanced cortical modulations are open for further inquiries, we discuss their potential role in processing execution errors and representing corrective or explorative activity. These representations are expected to contribute to the formation of internal models of the external actuator and their decoding may facilitate BMI improvement.

## Introduction

Brain Machine Interfaces (BMIs) hold promise for restoring motor functions in severely paralyzed patients [1]–[7]. State of the art BMIs take advantage of recent advances in electrophysiological techniques and neural decoding algorithms. Multi-electrode arrays facilitate simultaneous recordings from hundreds of neurons in multiple cortical areas. Movement related signals that modulate the activity of these neurons are extracted using neural decoding techniques and employed to control an external actuator. The BMI neural decoder includes free parameters that in typical BMI experiments with monkeys are determined from neural recordings during a training session with reaching movements. However, the requirement to produce similar movements *through* the BMI introduces a novel motor context, which may in turn affect the cortical modulations that drive the BMI.

During planning and execution of reaching movements, the modulations in the firing rate of cortical motor neurons reflect multiple motor, sensory, and cognitive variables [8]–[12]. Neural modulations that represent the direction and speed of the movement have been extensively studied during stereotypical reaching movements, and described computationally using tuning curves [13]–[16]. Recent BMI experiments indicate that neural tuning to movement direction [6] or velocity [7], [17] may change following BMI operation. However, changes in neuronal modulations beyond those related to movement kinematics have not been investigated.

The operation of a BMI presents a novel motor context in which the external actuator is controlled based on the predictions generated from a limited subset of neurons. During initial BMI operation, the movement of the actuator may deviate from the intended movement, and result in errors, as evident from the degradation in behavioral performance [7]. Here we addressed the effect of operating in this novel motor context on the nature of neuronal modulations in the motor cortex. Our spike-train analyses show that initial BMI operation was associated with increasing neuronal modulations, which were not merely associated with movement kinematics.

Firing-rate modulations are masked by neural noise, which hampers their unambiguous estimation form recorded spike-trains [18]. Averaging techniques that are applied to reduce the neural noise also diminish the effect of rate-modulating signals. Current rate-estimation methods focus on firing-rate modulations that are correlated with specific modulating signals, like the direction and speed of the movement. Such methods rely on identifying the relevant modulating signals and ignore potential contributions by other signals [19]. Instead of estimating the firing-rate, we focus in this paper on quantifying its variance, i.e., the variance of the neural activity that is associated with overall rate-modulations. This provided a scale for assessing the variance associated with specific modulating signals, which were estimated using linear regression. Using these tools we demonstrate that the variance associated with neuronal rate-modulations increased during initial BMI operation without a matching increase in the variance explained by the movement kinematics. Furthermore, the variance associated with neuronal modulations decreased with subsequent BMI training sessions. Possible hypothesis regarding the nature of these enhanced modulations are discussed to motivate further research.

## Methods

### Behavioral task and brain-machine interface operations

The BMI experiments were performed in two adult female monkeys (Macaca mulatta) and consisted of 10 BMI sessions for the first monkey and 23 for the second. The experiments are described in detail in [7] and briefly described here. Neural activity was recorded from *N_n_* = 100−300 neurons in multiple cortical areas including the primary motor cortex (M1), dorsal premotor cortex (PMd), supplementary motor area (SMA), and primary somatorsensory cortex (S1) in one monkey and medial intraparietal (MIP) of the posterior parietal cortex (PP) in the second monkey.

Each experimental session started with a training session in which the monkey controlled the position of a cursor on a computer screen by moving a hand-held pole (*pole control*), with the task of acquiring a randomly placed visual target within 5 sec to obtain a juice reward. A linear filter was trained to predict the velocity of the movement from the binned spike-counts of the recorded neurons. After training, the filter generated real-time predictions of the velocity, which were reproduced by the cursor and/or a robotic arm (*brain control*). The monkeys continued to move the pole after brain control started (*brain control with hand movements, BCWH*), but later assumed a stationary arm posture after the pole was taken away (*brain control without hand movements, BCWOH*). Lack of muscle activity during *BCWOH* was demonstrated by EMG measurement from wrist flexors, extensors and biceps. Performance accuracy diminished after the transition from pole- to brain-control, and after the monkeys stopped moving their arms. However, the BMI task performance improved with further training in all the control modes, clearly indicating that motor learning was involved.

### Percent Overall Modulation (POM)

**Figure 1:**
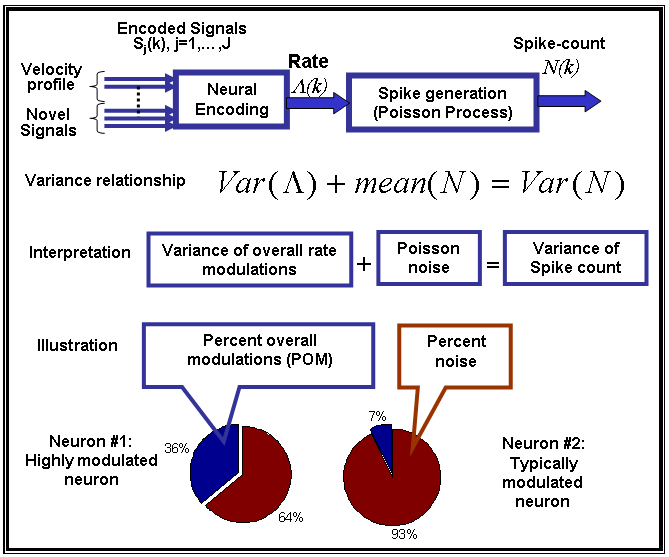
Inhomogeneous Poisson process and implied variance distribution.

Spike-trains can be considered as realizations of point processes [20], [21]. The number of spikes recorded in a bin of size *b, N_b_*, depends on the average bin-rate in that bin Λ*_b_*, which is modulated by the encoded signals, as depicted in Figure 1 (upper diagram). However this dependence is stochastic and the variance of the spike-count *Var*[*N_b_*] may differ from the variance of the bin-rate *Var*[Λ*_b_*]. While the variance of the spike-count can be measured directly, it is the variance of the bin-rate that is of interest here because it reflects signal modulations. In order to investigate these modulations, we defined the percent overall modulation (*POM*) as:
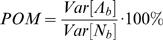
(1)Since the variance of the bin-rate cannot be measured directly, the *POM* cannot be estimated without further assumptions. However, instead of restricting the analysis to firing-rate modulations that involve specific modulating signals, we made only basic assumptions about the nature of the spikes trains. In particular, we assumed that the spike trains are realizations of *inhomogeneous* Poisson processes, which are the simplest point processes that can describe rate modulations. We further generalized the analysis to additive noise models whose variance is proportional to the mean.

The simplest point process, the *homogenous* Poisson process, is characterized by a constant rate and thus is inadequate for describing task-related firing rate modulations. Despite their constant rates, homogeneous Poisson processes generate spike trains with highly variable spike-counts, whose variance equals the mean, i.e., *Var*[*N_b_*] = *E*[*N_b_*]. Taking the homogeneous Poisson process as a model of neural activity with no modulations, we conclude that the resulting variance, which equals the mean rate, is attributed to the neural noise.

The *in*homogeneous Poisson process, which is characterized by time-varying rate that is independent of the history of the spike train, is the simplest point process that can describe firing rate modulations [20], [21], [22]. For inhomogeneous Poisson processes, the variance of the spike-count *Var*[*N_b_*] is related to the variance of the bin-rate *Var*[Λ*_b_*] according to *Var*[*N_b_*] = *Var*[Λ*_b_*]+*E*[*N_b_*] [20]. This relationship can be interpreted as a decomposition of the total variance in the binned spike-count into the variance of the underlying information bearing parameter, or bin-rate *Var*[Λ*_b_*], and the variance that would have occurred if *N_b_* was generated by a homogenous Poisson process, *E*[*N_b_*], i.e., the neural noise (Figure 1). Thus, the variance of the modulated bin-rate is the excess variance of the binned spike-count beyond the level expected from a homogeneous Poisson process. The resulting *POM* can be evaluated from the statistics of the binned spike-count as (Figure 1): 
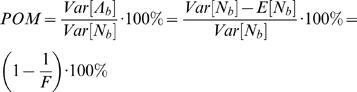
(2)where 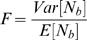 is the Fano factor [23], [22]. Given spike-trains of finite duration, we estimated the *POM* using the sample-mean and sample-variance of the binned spike-counts, instead of the mean and variance, respectively.

The *POM* analysis can be extended to cases where the binned spike-count include signal-dependent zero-mean noise, i.e., *N_b_* = Λ*_b_*+*e* [24]. The probability distribution of the noise *e* is assumed to be conditionally normal with signal dependent variance: *f*(*e*|Λ*_b_*)∼*N*(0,*γ* ^2^Λ*_b_*). Note that this model converges to the inhomogeneous Poisson process for large rates and *γ* = 1. Since for any signal level the error is zero mean, the variance of the spike counts is given by: *Var*[*N_b_*] = *Var*[Λ*_b_*]+*γ* ^2^ *E*[*N_b_*], as shown in the Supplemental text (Text S1). The resulting *POM* can be evaluated as: 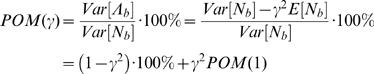
(3)where *POM*(1) is the *POM* of an inhomogeneous Poisson process (*γ* = 1) as defined in Equation (2). Thus, the results and conclusions based on the *POM* defined in Equation (2) can be easily extended for the general case of additive signal-dependent noise. In particular: 
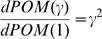
, so increasing/decreasing trends in estimated *POM* based on Equation (2) reflect increasing/decreasing trends in *POM*(*γ*).

### Significantly Modulated Neurons

The *POM* of spike-trains that are evoked by homogeneous Poisson processes is zero. However, when estimated from finite spike-trains, the sample-*POM* is randomly distributed and may take non-zero, either positive or negative, values. Under the homogeneous Poisson process assumption, the distribution of the sample-*POM*, estimated from a finite sample of *n* bins, is asymptotically normal with zero mean and variance of 2/*n*. We defined neurons as significantly modulated if their estimated *POM* indicated that the homogeneous Poisson process hypothesis could be rejected at 95% confidence level for at least one control mode (pole, BCWH, or BCWOH). For a typical sample of 12000 bins (20 minutes of an experiment with 100 msec bins), the standard deviation of the sample- *POM* is 1.3%. Note that neurons that are significantly modulated in only one or two control modes may exhibit zero of even negative sample-*POM* in the other mode(s) due to the limited length of the analyzed spike-train.

### Percent Velocity Modulation (PVM)

Velocity tuning is often determined in center-out reaching experiments, where the direction of movement is approximately constant [13], [16]. For general arm movements, this method was generalized to characterize the tuning of the neural activity to the velocity at a specific time lag [17]: 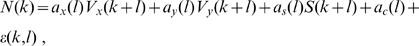
(4)where *k* is the index of the current time-bin, *N*(*k*) is the spike counts (in bins of 100msec), *V_x_* and *V_y_* are the x- and y-components of the velocity, 
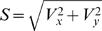
 is the speed, *l* is the relative lag (positive/negative *l* corresponds to rate-modulations preceding/ succeeding the velocity measurement, respectively), *a_x_*(*l*), *a_y_*(*l*) and *a_s_*(*l*) are the tuning parameters, *a_c_*(*l*) is a bias parameter, and *ε*(*k, l*) is the residual error. The coefficient of determination of the single-lag regression *R^2^(l)* quantifies the fraction of the variance in the neural spike-count that is attributed to, or explained by, the velocity at lag *l*. However, since the velocity values at different lags are correlated, the lag-by-lag analysis cannot be used to determine the fraction of the variance that is attributed to the spatio-temporal velocity profile. Hence, we further generalized the analysis to account for the tuning of the neural activity to the spatio-temporal velocity profile in the surrounding window according to: 
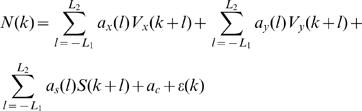
(5)where *ε*(*k*) is the residual error, and *L*_1_ and *L*_2_ are the number of preceding and succeeding lags in the velocity profile, respectively. We used *L*_1_ = *L*_2_ = *L* = 9 to include a 1900-ms window around the current bin. The regression in Equation (5) was evaluated using truncated Singular Value Decomposition to stabilize the solution despite the large condition number of the spatio-temporal velocity matrix (around 10^6^) [25], [26]. Truncation was performed at the singular value that preserved 95% of the variance in the velocity measurements.

The coefficient of determination of the spatio-temporal regression of Equation (5), *R^2^*, describes the fraction of the variance in the binned spike-count that is linearly related to variations in the spatio-temporal velocity profile in the surrounding window. Expressed as a percentage, *R^2^* is referred to as the percent velocity-related modulation, or *PVM*.

### Percent Kinematics Modulation (PKM)

The above analysis was further extended to include other kinematics variables that may modulate the neural activity, including the position and magnitude of acceleration: 
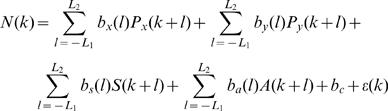
(6)Where *P_x_* and *P_y_* are the x- and y-components of the position, and 
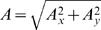
 is the magnitude of the acceleration vector whose x-component is given by *A_x_*(*l*) = *V_x_*(*l*)−*V_x_*(*l*−1) = *P_x_*(*l*)−2*P*(*l*−1)+*P_x_*(*l*−2) and y-component given by the corresponding expression. Note that the velocity and acceleration vectors are implicitly included in Equation (6) as the first and second order differences between the positions in consecutive lags.

The coefficient of determination of the spatio-temporal regression in Equation (6) was expressed as a percentage and referred to as the percent kinematics modulation, or *PKM*.

### Variations in percent modulations

In order to evaluate the variations in the percent modulations (i.e., *POM, PVM* or *PKM*) within and across different control modes and training sessions, we estimated their values for each neuron in 2-minute non-overlapping intervals. Within each control mode, the *POM, PVM* and *PKM* of individual neurons were determined by averaging across all the relevant 2-minute intervals. The ensemble-*POM*, ensemble-*PVM* and ensemble-*PKM* in a specific 2-minute interval were determined by averaging across all the significantly modulated neurons. Comparisons between control modes and training sessions were based on the mean-*POM*, mean-*PVM* and mean-*PKM*, which were computed by averaging the corresponding ensemble values across all the intervals in the same control mode.

### Principal Component Analysis

Principal component analysis (PCA) is a standard technique for discovering the dimensionality of a data set and decomposing it into uncorrelated components [27], [28]. The sequences of spike-counts from *N_n_* neurons is transformed linearly (with unit norm weight vectors) into *N_n_* principal components, which are uncorrelated with each other and have extreme variance values. Since the principal components in this application are weighted sums of the recorded spike-counts they may be referred to as “principal-neurons”.

Specifically, we computed the *N_n_*×*N_n_* sample-correlation matrix of the normalized spike-counts of the *N_n_* neurons, whose *i-j* element is the sample-correlation between the normalized spike-counts *N_i_* and *N_j_* of the *i-th* and *j-th* neurons, recorded during the relevant part of the experiment. The variance of the principal-neurons was determined by the eigen-values 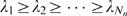 of the sample-correlation matrix. In particular, *λ*_1_ is the maximum variance of any weighted-combination of the recorded spike-counts with a unit norm weight vector. The normalized eigen-value 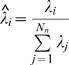 describes the fraction of the variance in the original data that is captured by the *i-th* principal-neuron. Expressed as a percentage, it defines the percent of the total variance in the neural ensemble carried by the *i-th* principal-neuron.

## Results

**Figure 2:**
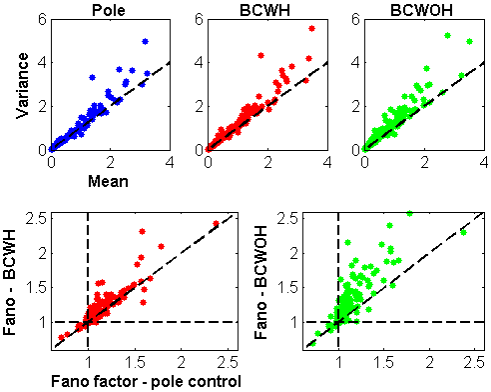
Firing rate statistics for *N_n_* = 183 units recorded in one session. Mean-variance relationship computed based on 100-ms bins (top panels); and scatter plots of Fano factor in brain versus pole control (bottom panels). BCWH–Brain control with hand movements, BCWOH–Brain control without hand movements.

The neural activity of most of the cortical neurons was more variable during brain control than during pole control. Generally, rate variability was higher than would be predicted by a homogeneous Poisson process, as indicated by variance that exceeded the mean (Figure 2, top panels). The variance of the binned spike-count (in 100-ms bins) exceeded the mean spike-count for most of the *N_n_* = 183 neurons recorded in this session, (83%, 88% and 92% during pole control, BCWH and BCWOH, respectively). This excess variability was also evident in the ratio of the variance to the mean (the Fano factor), which was found to be mostly above 1.0 (Figure 2, bottom panels). Most importantly, the transition to brain control resulted in even higher excess variability and larger Fano factor for most of the neurons (78% and 87% of the recorded cortical neurons during BCWH and BCWOH, respectively) as evident from the scatter plots of Figure 2.

### Percent Overall Modulation

**Figure 3:**
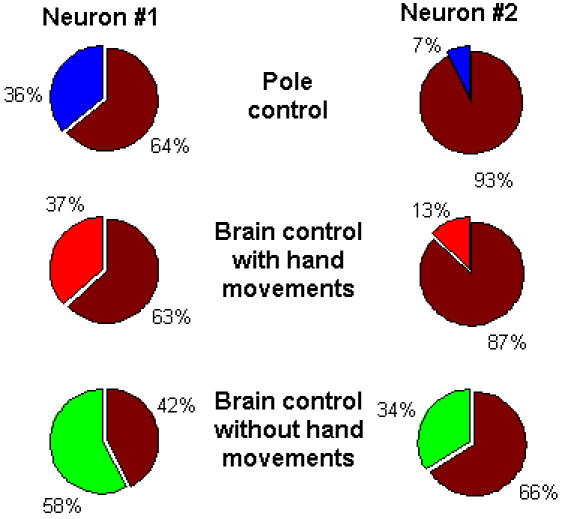
Percent Overall Modulation (*POM*) in pole control, BCWH and BCWOH for two representative M1 units.

The *percent overall modulation* (*POM*), defined in Equation (1), represents the percentage of the variance of the binned spike-count that is attributed to rate modulations (Figure 1, Methods). The pie-plots in Figure 3 illustrate the distribution of the variance in the spike-counts recorded from two M1 neurons in different control modes, based on the *POM* computed using Equation (2) (Methods). During pole control (Figure 3, top pies), rate modulations accounted for only 7% for the typical neuron depicted on the right and 36% for a highly modulated neuron depicted on the left. The contribution of rate-modulations to the variance of the neural activity was more significant in brain control with and without hand movements, accounting, respectively, for 13% and 34% of the variance for the typical neuron (Figure 3, right middle and bottom pies) and 37% and 58% for the highly modulated neuron (Figure 3, left middle and bottom pies). These examples indicate that *POM* was higher in brain control than in pole control.

**Figure 4:**
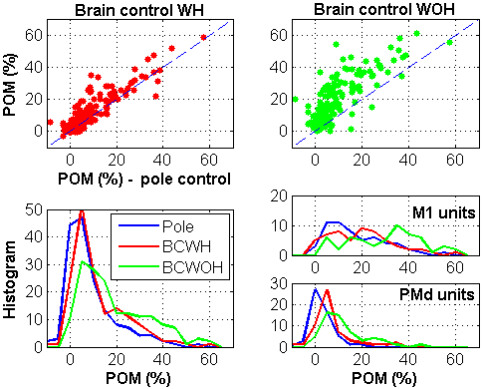
Percent Overall Modulation (*POM*) for *N_sig_* = 160 significantly modulated units recorded in the same session analyzed in Figure 2. Top: Scatter plot of *POM* in brain versus pole control. Bottom: distribution of POM of all significantly modulated units (left) and those in M1 (right upper, *N_sig_* = 56) and PMd (right lower, *N_sig_* = 55).

Figure 4 demonstrates the same trend for all the significantly modulated neurons. Insignificantly modulated neurons, i.e., neurons with little or no rate modulation in all the control modes (Methods), were considered irrelevant for task performance, and were excluded from further analysis. During the session analyzed in Figure 4, 87% of the 183 recorded neurons exhibited significant modulations in at least one control mode. Most of the significantly modulated neurons exhibited higher *POM* during brain control than during pole control, as evident in the top panels of Figure 4 (78% and 91% of the significantly modulated neurons in BCWH and BCWOH, respectively, Table 1). For few neurons the estimated *POM* during pole control was even negative (possibly due to the finite length of the spike-train, see Methods) and became positive only during BCWH or BCWOH. Thus, the top panels in Figure 4 depict the changes in the firing rate statistics expressed in Figure 2 in terms of the *POM.*

**Table 1:** Number of neurons with increasing POM (*POM_inc_*) and increasing variance (*Var_inc_*), during the sessions depicted in Figure 4, and 5, out of the *N_sig_* significantly modulate neurons.

Transition		All	PMd	M1	S1	SMA
**Figure 4** **, ** **5**						
*N_n_* = 183	*N_sig_*	160(*)	**55**	**56**	**33**	**13**
**Pole → BCWH**	*POM_inc_*	**125**	**48**	**35**	**28**	**12**
	% of *N_sig_*	**78%**	**87%**	**62%**	**85%**	**92%**
	*Var_inc_*	**97**	**44**	**33**	**16**	**4**
	% of *POM_inc_*	**78%**	**92%**	**94%**	**57%**	**33%**
**BCWH → BCWOH**	*POM_inc_*	**139**	**46**	**51**	**31**	**8**
	% of *N_sig_*	**87%**	**84%**	**91%**	**94%**	**62%**
	*Var_inc_*	**72**	**39**	**23**	**3**	**7**
	% of *POM_inc_*	**51%**	**85%**	**45%**	**10%**	**87%**
**Pole → BCWOH**	*POM_inc_*	**145**	**51**	**50**	**32**	**10**
	% of *N_sig_*	**91%**	**93%**	**89%**	**97%**	**77%**
	*Var_inc_*	**104**	**50**	**37**	**12**	**5**
	% of *POM_inc_*	**72%**	**98%**	**74%**	**37%**	**50%**
**Figure 6**						
*N_n_* = 185	*N_sig_*	169(*)	**59**	**56**	**36**	**15**
**Pole → BCWOH**	*POM_inc_*	**155**	**56**	**50**	**35**	**11**
	% of *N_sig_*	**92%**	**95%**	**89%**	**97%**	**73%**
	*Var_inc_*	**116**	**52**	**36**	**23**	**4**
	% of *POM_inc_*	**75%**	**93%**	**72%**	**66%**	**36%**

(*) Including neurons in ipsilateral M1, which are too few to analyze as a group.

The distributions of the *POM* of the significantly modulated neurons during brain control (BCWH and BCWOH) differed significantly from the distribution of the *POM* during pole control (Wilcoxon rank sum test, *p* = 1.2% and *p* = 10^−10^% for BCWH and BCWOH, respectively) as shown in the bottom left panel of Figure 4. The mean *POM* (±std) in the different modes were 8.8%±10.7%, 12.2%±11.7%, and 18.8%±14.4% for pole control, BCWH, and BCWOH, respectively. The corresponding distributions for M1 and PMd neurons are shown in the right bottom panels of Figure 4.

**Figure 5:**
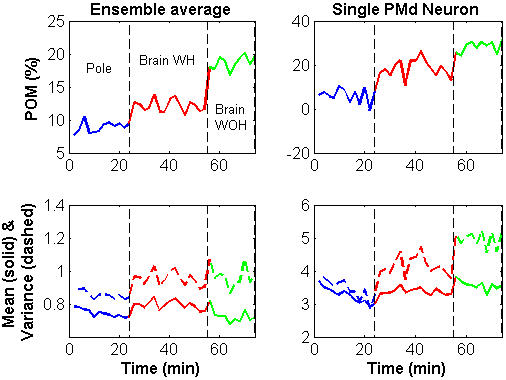
Variations in *POM* (top) and spike-count statistics (bottom) during the same experimental session analyzed in Figure 4 (ensemble-average–left panels; single PMd unit–right panels). The *POM* and spike-count statistics were computed in 2-minute non-overlapping intervals and averaged across all the *N_sig_* = 160 significantly modulated neurons to obtain the ensemble-average.

Variations in *POM* during that session are shown in Figure 5 for the ensemble-mean (top left) and for a typical PMd neuron (top right). The ensemble-*POM* is the average *POM* over all the significantly modulated neurons (Methods). The figure indicates that the ensemble-*POM* remained relatively stable in each mode of operation, but changed abruptly after the transition to brain control and especially after the transition to brain control without hand movements.

### *POM* and firing rate statistics

The underlying changes in the spike-count statistics (bottom panels of Figure 5) indicate that the ensemble*-POM* increased mainly due to an increase in the variance of the spike-count, which was not matched by the change in the mean spike-count. Indeed, most of the neurons (78%) whose *POM* was higher in BCWH than in pole control, exhibited also a larger variance, especially neurons in PMd (92%) and M1 (94%), as summarized in Table 1.

The transition from BCWH to BCWOH resulted in further increase in *POM* for most of the significantly modulated neurons (87%). This increase resulted from a combination of increasing variance and decreasing mean rate. Increasing *POM* from BCWH to BCWOH was usually associated with increasing variance, especially for PMd neurons (85%, Table 1), as shown for a typical PMd neuron in Figure 5 (bottom right). Furthermore, considering the overall transition from pole control to BCWOH, increasing *POM* was associated with increasing variance for most of the neurons (72%); especially among PMd (98%) and M1 (74%) neurons (Table 1). Thus, the overall increase in *POM* from pole control to BCWOH is attributed mainly to increasing variance.

**Figure 6:**
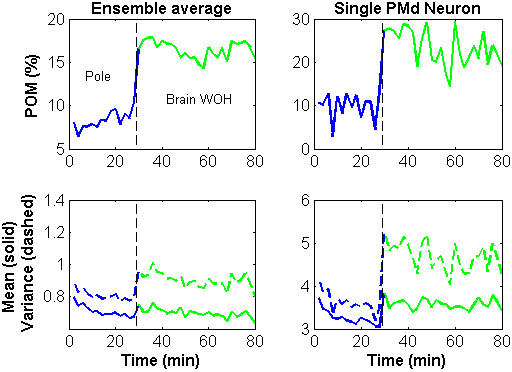
Variations in *POM* (top) and spike-count statistics (bottom) during an experimental session with direct transition form pole control to BCWOH (ensemble-average–left panels; single PMd unit–right panels). The *POM* and spike-count statistics were computed in 2-minute non-overlapping intervals and averaged across all the *N_sig_* = 169 significantly modulated neurons to obtain the ensemble-average.

Direct transition from pole control to BCWOH was tested in later sessions in which the joystick was removed immediately after the pole control epoch. The changes in the *POM* and the firing rate statistics during such a session are depicted in Figure 6. The large increase in the *POM* after the transition to BCWOH (top panels, ensemble average–left, a typical PMd neuron–right) is related mainly to increasing variance (bottom panels, respectively). Indeed, most (75%) of the neurons whose *POM* was higher in BCWOH than in pole control, exhibited also a larger variance, especially neurons in PMd (93%), and M1 (72%) (Table 1).

### *POM* in different cortical areas

**Figure 7:**
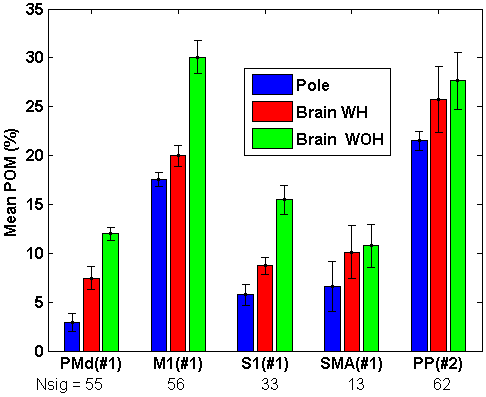
Mean-*POM* of the N_sig_ significantly modulated units in different cortical regions during different control modes in a single session with each monkey. Full bars mark the mean values across all the 2-minute intervals in the same control mode, and error bars represent the standard deviation. M1–primary motor cortex, PMd–dorsal premotor cortex, S1–primary somatosensory cortex, SMA–supplementary motor area, PP–Posterior Parietal Cortex (medial intraparietal).

The mean-*POM*, computed as the mean of the ensemble-*POM* across each control mode (Methods), was higher in brain control than in pole control for all cortical areas examined from both monkeys (Figure 7, Table 2). The standard deviation of the ensemble-*POM* during each control mode is marked by an error bar (reflecting variations in the ensemble-*POM* across time). The change in the mean-*POM* from pole control to either BCWH or BCWOH was higher than the respective standard deviations in most of the recorded cortical areas (except for the limited group of SMA neurons).

**Table 2:** Mean-*POM* and Mean-*PVM* of neurons in different brain regions during each control mode. The standard deviations reflect variations in the ensemble-statistics across time.

Mean±std		PMd	M1	S1	SMA	PP
	Monkey	#1	#1	#1	#1	#2
	*N_sig_*	55	56	33	13	62
**POM (%) ** **Fig 7**	**Pole**	**2.9±0.9**	**17.5±0.7**	**5.7±1.0**	**6.6±2.3**	**21.5±1.0**
	**BCWH**	**7.5±1.1**	**20.0±1.1**	**8.7±0.9**	**10.1±2.7**	**25.7±3.3**
	**BCWOH**	**12.0±0.7**	**30.1±1.6**	**15.5±1.5**	**10.7±2.9**	**27.6±2.9**
**PVM (%) ** **Fig 10**	**Pole**	**1.2±0.2**	**7.2±0.5**	**3.7±0.7**	**4.3±1.4**	**7.3±0.6**
	**BCWH**	**2.3±0.3**	**6.4±0.5**	**3.3±0.5**	**4.2±0.9**	**9.8±2.6**
	**BCWOH**	**2.1±0.3**	**5.1±1.1**	**2.7±1.0**	**2.4±0.4**	**6.9±3.6**
**PKM (%)**	**Pole**	**1.3±0.2**	**8.0±0.7**	**4.2±0.8**	**4.4±1.3**	**7.2±0.6**
	**BCWH**	**2.6±0.4**	**7.1±0.5**	**3.9±0.5**	**4.3±0.9**	**9.2±2.6**
	**BCWOH**	**2.4±0.3**	**5.8±1.2**	**3.2±1.1**	**2.6±0.5**	**7.2±3.8**

### Variance distribution in principal neurons

**Figure 8:**
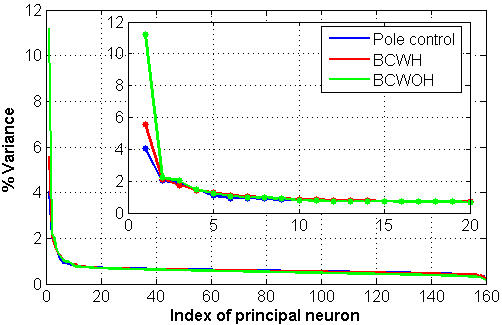
Percent variance carried by the different principal neurons during the same session analyzed in Figure 5. The analysis decomposed the variance carried by the ensemble of the significantly modulated neurons recorded from all brain regions. Inset: zoomed-in version of the same plot for the initial 20 principle neurons.

The *POM* analysis decomposed the variance of the spike-counts of individual neurons into the variance associated with rate modulations and the variance associated with neural noise (Figure 3). At the ensemble level, principal component analysis (PCA, Methods) was used to decompose the total variance of the neural activity into uncorrelated principal components, or “principal neurons”. Figure 8 depicts the percent variance carried by the different principal neurons during the same session analyzed in Figures 4, 5 and 7. The percent variance dropped sharply for the first few principal neurons and remained relatively constant thereafter. In an ideal case, when the noise is generated from independent identically distributed random processes, the percent variance of the background noise is constant. Thus, the relatively constant variance level beyond the initial 5–15 principal neurons can be attributed mainly to the neural noise. In contrast, the excess variance of the initial principal neurons above the background level reflects correlated activity among the different neurons and can be attributed mainly to rate-modulations by common modulating signals.

The decomposition of the variance of the ensemble activity into principal neurons extends the single-neuron based *POM* analysis, and suggests that the modulating signals responsible for the *POM* were highly correlated. Indeed, the accumulated excess variance in the initial principal neurons (Figure 8) was comparable to the average *POM* indicated in Figure 5, and small compared with the accumulated variance of the background noise level. Furthermore, as seen from Figure 8, the variance of the first principal neuron, i.e., the maximum variance of any weighted sum of the neural activity (with unit norm weight vector), was higher during brain control than during pole control. This indicates that the variance of the modulating signals, which contributed to correlated neural activity, was higher during brain control than during pole control.

### Percent velocity modulation

The contribution of the velocity of movement (both the velocity-vector and the speed) to the variability in the neural activity, was evaluated using the regression between the spike counts in 100-ms bins and the velocity profile during the surrounding 1900-ms window (Methods, Equation (5)). Spike trains evoked during pole control were related to the hand velocity, while spike trains evoked during BCWOH were related to the cursor velocity. The analysis for BCWH accounted for the correlation with both the hand and cursor velocities. The coefficient of determination, *R^2^*, of the regression quantifies the fraction of variance in binned spike-counts that is attributed to the velocity profile. Expressed as a percentage, the coefficient of determination is referred to as the percent velocity-related modulation (*PVM*, Methods).

We also extended the analysis to include all the kinematics variables, i.e., the time course of the position (which implicitly includes the time course of the velocity and acceleration), speed and magnitude of the acceleration, in the surrounding 1900-ms window, as described by Equation (6) (Methods). The resulting coefficient of determination was expressed as a percentage and referred to as percent kinematics modulation (*PKM*, see Methods). Overall, the results for the *PKM* were similar to the *PVM*, and are stated only briefly for completeness.

**Figure 9:**
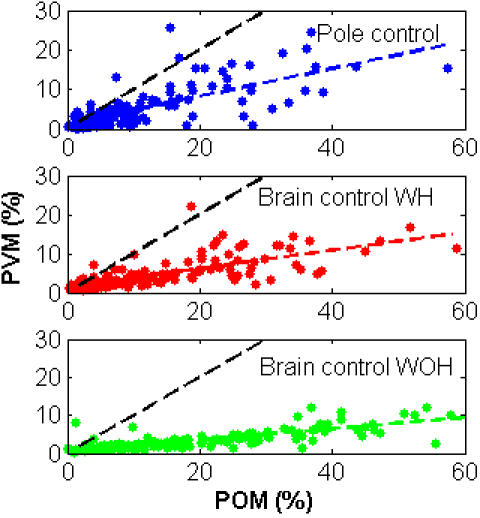
Correlation between *PVM* and *POM* for all the N_sig_ = 160 significantly modulated neurons recorded in one session. Coloured dashed lines mark the linear regression lines. Black dashed lines mark the diagonal unit-slope lines.

*PVM* was significantly correlated with *POM* (p<10^−10^), as shown in Figure 9, with coefficients of correlation of 0.72, 0.68 and 0.79 for pole control, BCWH and BCWOH, respectively (0.72, 0.71 and 0.81, respectively for *PKM* versus *POM*). This strong correlation implies that the activity of cortical neurons, which exhibited larger rate modulations, was, in general, better correlated with the velocity profile, and can thus contribute more to its prediction. In contrast, there was no significant correlation between *POM*, or the increase in *POM*, and the magnitude of the decoding weights used in the BMI filter.

While *PVM* was strongly correlated with *POM*, the slope of the linear relationship was only 0.31, 0.21 and 0.12, for pole control, BCWH and BCWOH, respectively (0.35, 0.23 and 0.14, respectively for *PKM* versus *POM*). The smaller than unit slopes indicate that only a small fraction of the modulations was correlated with the velocity profile. Most importantly, the slopes of the *PVM-POM* or *PKM-POM* relationships were smaller in brain control compared to pole control. This suggests that modulating signals, which were not correlated with the kinematics of the movement, had a larger effect on the *POM* during brain control than during pole control.

**Figure 10:**
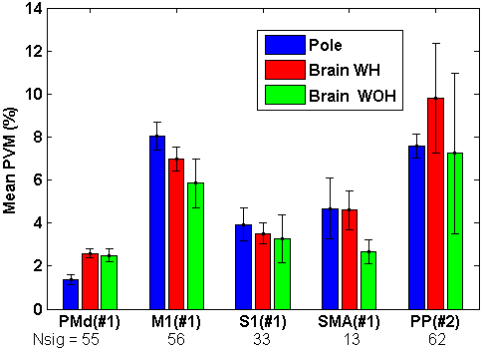
Mean-*PVM* of the N_sig_ significantly modulated units in different cortical regions during different control modes in the same sessions as Figure 7. Full bars mark the mean values across all the 2-minute intervals in the same control mode, and error bars represent the standard deviation. M1–primary motor cortex, PMd–dorsal premotor cortex, S1–primary somatosensory cortex, SMA–supplementary motor area, PP–posterior parietal cortex (medial intraparietal).

The mean-*PVM* in most cortical regions did not increase significantly after switching to brain control, as indicated in Figure 10. Thus the mean-*PVM* did not follow the significant and large increase in the mean-*POM* shown in Figure 7. While the mean-*POM* of M1 neurons, for example, increased from 17.5±0.7% in pole control to 20.0±1.1% in BCWH and 30.1±1.6 in BCWOH, the mean-*PVM* decreased from 7.2±0.5% to 6.4±0.5% and 5.1±1.1% (Table 2). The mean-*PKM* (Table 2) was at the most 0.8% higher than the mean-*PVM*, and decreased from 8.0±0.7% for M1 neurons during pole control to 7.1±0.5% and 5.8±1.2%, during BCWH and BCWOH, respectively. Since the increase in *POM* was not matched by increasing *PVM* or *PKM*, the higher neuronal rate modulations observed during brain control cannot be explained only by increased modulations due to the kinematics of the movement.

### Effect of training

**Figure 11:**
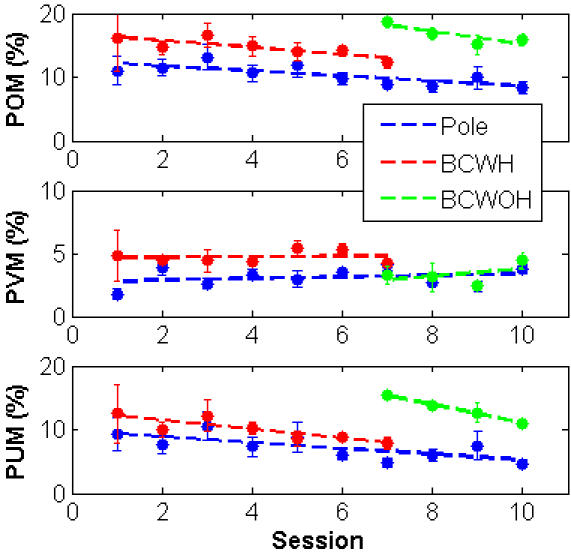
Effect of training: percent overall modulation *POM*, percent velocity modulation *PVM* and percent untagged modulation *PUM* of the N_sig_ significantly modulated neurons during all the 10 BMI sessions with monkey #1. N_sig_ = 156, 130, 142, 142, 150, 140, 160, 166, 174 and 165. Initial sessions included BCWH while later sessions included only BCWOH.

In all the experimental sessions, with both monkeys, the *POM* was always higher in brain control than in pole control, as demonstrated in Figure 11 (top panel). Furthermore, in all the control modes, the *POM* decreased gradually with training. These trends were statistically significant in pole control and BCWH (p<0.05). In contrast, the mean-*PVM* remained approximately the same, and even increased, with training (Figure 11, middle panel). The difference between the two, i.e., *POM* minus *PVM*, is depicted in the bottom panel of Figure 11, and exhibited statistically significant decreasing trends in all the control modes (p<0.02). Similar results were obtained when considering the mean-*PKM*, which accounts for modulations by the kinematics of the movement. Thus, the changes in *POM* during BMI training seem to reflect mainly changes in untagged modulations not correlated with the kinematics of the movement.

The observed trends in the mean-*POM* and in the percent untagged modulations mirrored the trend in task performance [7]. Behavioral performance, quantified by either the time to reach the target or the success-rate, deteriorated after switching to brain control, but improved gradually with training. Thus, in general, the *POM* and the percent untagged modulations increased as the behavioral performance degraded and decreased as the behavioral performance improved.

## Discussion

Our analyses indicate that cortical neurons that are used to control a BMI modulate their activity more intensely during brain control than during pole control. The enhanced modulations were evident in increased variability of the binned spike-counts beyond the level expected from homogeneous Poisson processes. We quantified this excess variability using the *POM*, which described the overall contribution of modulating signals to the variance of the binned spike-counts of individual neurons. The method for estimating the *POM* was based on the assumption that the spike trains were realizations of inhomogeneous Poisson processes. Nevertheless, we showed that the analysis is relevant for a broader class of spike-trains with signal dependent additive noise. Thus, the observed changes in the estimated-*POM* are indicative of changes in the percent variance attributed to overall modulations in the underlying rate under a wide range of assumptions.

The excess variance attributed to rate modulations was also quantified using principal component analysis (PCA). The accumulated excess variance in the initial principal neurons, above the background noise level, was similar to the average *POM*. Furthermore, the variance of the 1^st^ principal neuron, which represented the variance of the most correlated linear-component of the neural activity, was higher in brain control than in pole control. Thus, the PCA indicated that the neural activity during brain control included a larger component of correlated activity compared to pole control, in support of the conclusions from the *POM* analysis.

We also evaluated the percent variance of the binned spike-counts that can be attributed to velocity or kinematics modulations, and defined it as the *PVM* or *PKM*, respectively. Comparing the changes in *POM* with the changes in *PVM* or *PKM* revealed that the enhanced modulations cannot be attributed solely to velocity or even kinematics modulations. Furthermore, as training progressed, and the monkeys became more proficient in operating the pole and the BMI, the *POM* decreased while the *PVM* and *PKM* remained relatively constant. The observed trend in *POM* during BMI operation paralleled the effect on behavioral performance, which degraded during initial BMI operation and improved with subsequent training.

The firing rate modulations were especially strong when the monkeys controlled the cursor without moving their arms, although the neural signals that would be related to the movement of the arm were irrelevant in that mode. The exact source of these extra firing modulations cannot be assessed using the current BMI experiments, since only the movement kinematics was measured directly. However, the results motivate some hypotheses, as detailed below, which would be explored in future investigations.

The observed enhancement in neuronal rate-modulations may result from internal representation and processing of prediction- and execution-errors, which intensify when starting to operate the BMI and weaken gradually with subsequent training. Prediction- and execution-errors [29], [30] are prevalent in novel behavioural contexts, possibly due to lack of appropriate kinematics or dynamic internal models [31]–[36]. The motor system may correct the execution-errors on-line using feedback control [37], [38] or initiate explorative activity to learn the new environment. Thus, the enhanced activity in different cortical areas may reflect the larger prediction and execution errors during initial BMI operation, and the resulting feedback and/or explorative activity.

During the BMI experiments, the internal models are expected to adapt to improve BMI operation and capture its input/output relationship. This may explain the changes in the tuning curves of the recorded neurons [6], [17], and the improvement in task performance with training [7]. Regardless of the nature of the adaptation mechanism, the improved task performance implies that execution errors diminished with training. Thus, our hypothesis is supported by the observed reduction in *POM* with training, despite the relatively invariant *PVM*.

Our hypothesis can also explain the relatively moderate increase in *POM* when switching to BCWH, compared with the higher increase when switching to BCWOH. During BCWH, both the visual and proprioceptive feedbacks were relevant but provided conflicting error signals: the visual feedback indicated that the cursor was deviating from the desired trajectory, while the proprioceptive feedback indicated that the arm was following the desired trajectory. Under these conflicting error signals, the response would be attenuated and result in only moderately higher *POM*. During BCWOH, only the visual feedback was relevant and could trigger a full response, and thus an even higher *POM*.

Different cortical areas, including the ones studied here, have been shown to be involved in the different computational aspects of sensorimotor adaptation. Studies of prism adaptation demonstrated that the dorsal premotor area (PMd) plays an important role in on-line error corrections [39], [40], [41]. Remapping, which facilitates adaptation, seems to occur in the posterior parietal (PP) cortex [39]. The primary motor area (M1) is involved in both motor performance and the acquisition of new motor skills [42], [43], [44]. The supplementary motor area (SMA) is strongly involved in motor planning but is also involved in motor execution and seems to represent mainly the dynamics of the movement [45].

Our results are consistent with the reported role of the different cortical areas in on-line error-correction. In line with the role of PMd neurons in error correction, the increased execution error during initial BMI operation should increase the modulation of their activity. Indeed our results indicate that the *POM* of PMd neurons was significantly higher during brain control than during pole control (Figure 5 and Figure 6 right columns, Figure 7). Thus, the increased modulations of PMd neurons may reflect the corrective response of an internal feedback controller. Increased modulations in PMd are expected to result in increased modulations in M1, in agreement with the observed change in the *POM* of M1 neurons. Additionally, the increased modulations in S1 neurons may result from proprioceptive prediction errors.

Depending on the nature of the motor response, the enhanced neural modulations may encode only the magnitude or also the direction of the errors. Explorative activity might be generated based solely on the magnitude of the prediction or execution error. In contrast, feedback-corrections require also the representation of the direction of the error. In either case the enhanced modulations are expected to carry viable information during initial BMI operation that could be used for its improvement. Future experiments would be directed at exploring whether and how the enhanced modulations are tuned to prediction and execution errors.

Our hypothesis suggests that long-term use of a BMI would lead to its incorporation into internal models that would facilitate the accurate operation of the external actuator as if it was a natural limb. Exploring the nature of the enhanced modulations would facilitate the development of efficient BMIs and would provide further insight into the mechanism of adaptive skill acquisition.

## Supporting Information

Text S1 Variance analysis: variance relationship in signal dependent additive noise
(0.03 MB DOC)
