# Supplementary material for: Cortical Modulations Increase in Early Sessions with Brain-Machine Interface
Source: PLoS One. 2007 Jul 18;2(7):e619. doi: 10.1371/journal.pone.0000619 (PMC1919433; doi:10.1371/journal.pone.0000619)
Supplement: Text S1 — Variance analysis: variance relationship in signal dependent additive noise (0.03 MB DOC) [file pone.0000619.s001.doc]

**Variance relationship in signal dependent additive noise**

For large rates the Poisson process can be approximated by a Normal distribution with probability density function $f(N_{b})=N(\Lambda_{b},\Lambda_{b})$. This can be decomposed into a signal and signal-dependent noise term as:$N_{b}=\Lambda_{b}+e$, where the probability distribution of the noise is conditionally normal with signal dependent variance: $f(e|\Lambda_{b})\sim N(0,\Lambda_{b})$. Here we extend this model to consider cases where the variance of the noise is proportional, but not necessarily equal to the signal, i.e., where $f(e|\Lambda_{b})\sim N(0,\gamma^{2}\Lambda_{b})$.

As the signal changes with time so does the variance of the noise and the resulting spike-counts. The variance of the spike counts is given by:

$$\begin{matrix} Var[N_{b}] =E[\left(N_{b}-E[N_{b}]\right)^{2}]=E[\left(N_{b}-E[\Lambda_{b}]\right)^{2}]=E[\left(\Lambda_{b}+e-E[\Lambda_{b}]\right)^{2}] \\ =E[\left(\Lambda_{b}-E[\Lambda_{b}]\right)^{2}]+E[e^{2}]+2E[\left(\Lambda_{b}-E[\Lambda_{b}]\right)e] \end{matrix}$$

Considering each of these terms we have:

$$E[\left(\Lambda_{b}-E[\Lambda_{b}]\right)^{2}]=\mathrm{var}(\Lambda_{b})$$

$$E[e^{2}]=\int f(\Lambda_{b})\left(\int f(e|\Lambda_{b})e^{2}de\right)d\Lambda_{b}=\int f(\Lambda_{b})\gamma^{2}\Lambda_{b}d\Lambda_{b}=\gamma^{2}E[\Lambda_{b}]$$

Finally, since for any signal level the error is zero mean,

$$E[\left(\Lambda_{b}-E[\Lambda_{b}]\right)e]=\int f(\Lambda_{b})\left(\Lambda_{b}-E[\Lambda_{b}]\right)\left(\int f(e|\Lambda_{b})ede\right)d\Lambda_{b}=0$$

Thus, the variance of the binned spike counts is given by: $Var[N_{b}] =Var[\Lambda_{b}]+\gamma^{2}E[\Lambda_{b}]=Var[\Lambda_{b}]+\gamma^{2}E[N_{b}]$.
